# High Functionalization of 5-Nitro-1*H*-imidazole Derivatives: The TDAE Approach

**DOI:** 10.3390/molecules16086883

**Published:** 2011-08-12

**Authors:** Thierry Juspin, Laura Zink, Maxime D. Crozet, Thierry Terme, Patrice Vanelle

**Affiliations:** Laboratoire de Pharmaco-Chimie Radicalaire, Faculté de Pharmacie, Universités d’Aix-Marseille I, II et III – CNRS, UMR 6264: Laboratoire Chimie Provence, 27 Bd Jean Moulin, 13385 Marseille Cedex 05, France

**Keywords:** TDAE, 5-nitro-1*H*-imidazole, arylethanol, carbonyl derivatives

## Abstract

We report herein the synthesis of substituted 2--[4-(1,2-dimethyl-5-nitro-1*H*-imidazol-4-yl)phenyl]-1-arylethanols, ethyl 3-[4-(1,2-dimethyl-5-nitro-1*H*-imidazol-4-yl)-phenyl]-2-hydroxypropanoate and 2-[4-(1,2-dimethyl-5-nitro-1*H*-imidazol-4-yl)benzyl]-2-hydroxy-acenaphthylen-1(2*H*)-one from the reactions of 4-[4-(chloromethyl)phenyl]-1,2-dimethyl-5-nitro-1*H*-imidazole with various aromatic carbonyl and α-carbonyl ester derivatives using tetrakis(dimethylamino)ethylene (TDAE) methodology.

## 1. Introduction

The 5-nitroimidazole scaffold is well-known for displaying major anti-infectious activities [1,2,3,4,5,6]. Several 5-nitroimidazole-containing active principles such as metronidazole, secnidazole and ornidazole are commonly used in medecine. These chemotherapeutic agents inhibit the growth of both anaerobic bacteria and some anaerobic protozoa [7]. Nowadays, the most clinically used drug-compounds for the treatment of both infections caused by protozoa such as *Trichomonas vaginalis*, *Entamœba histolytica*, *Giardia intestinalis* and infections induced by anaerobic bacteria is metronidazole. However, the 5-nitroimidazoles have been found to possess a high mutagenic activity in prokaryotic micro-organisms. A nitroimidazole possessing good pharmacological activities with no mutagenicity [8] would be of great interest, not only from a safety point of view, but would also provide a basis for further investigations on the mechanism(s) involved in their mutagenicity. Moreover, emergence of metronidazole-resistant *Trichomonas vaginalis* has resulted in decreased success of current therapies [9,10]. These refractory cases are usually treated with higher doses of metronidazole, which leads in turn to an increase in the occurrence of side effects [10,11], so alternative curative therapies are needed. 

In continuation of our program directed towards the study of electron transfer reactions in heterocyclic series [12,13,14], we recently investigated S_RN_1 (unimolecular radical nucleophilic substitution) reactions of 4(5)-[4-(chloromethyl)phenyl]-1,2-dimethyl-5(4)-nitro-1*H*-imidazoles, involving long distance (six bonds) between the electron-withdrawing and leaving groups (LD-S_RN_1). The nitronate anions reacted by the substitution at the chloromethyl group and the reaction was very probably mediated by a S_RN_1 mechanism [15].

Tetrakis(dimethylamino)ethylene (TDAE) is a reducing agent which reacts with halogenated derivatives to generate an anion under mild conditions via two sequential transfers of one electron [16,17,18]. We have shown that from *o*- or *p*-nitrobenzyl chloride, TDAE could generate a nitrobenzyl carbanion which is able to react with various electrophiles [19]. Since 2003, and using this strategy, we have developed several reactions between nitrobenzylic, heterocyclic or quinonic substrates and a series of carbonylated electrophiles such as aldehydes [19,20,21,22], ketones [19,20,21,22], α-ketoesters [23,24,25], α-ketolactams [26], α-diketones [27,28] and diethyl ketomalonate [23,24,25] leading to the corresponding alcohol adducts. In continuation of our program directed toward the study of electron transfer reactions of bioreductive alkylating agents and the preparation of new and potentially safer nitroimidazoles, we report herein the synthesis of some highly functionalized 5-nitro-1*H*-imidazoles from the reaction of 4-[4-(chloromethyl)phenyl]-1,2-dimethyl-5-nitro-1*H*-imidazole with various aromatic carbonyl and α-carbonyl ester derivatives using TDAE methodology.

## 2. Results and Discussion

**Scheme 1:**
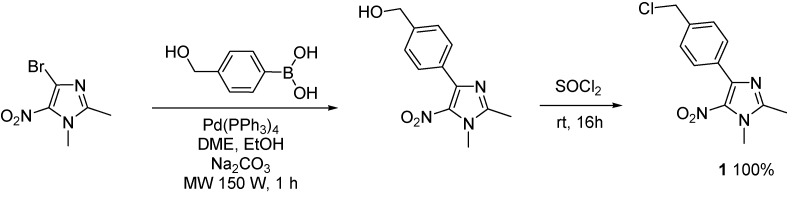
Preparation of 4-[4-(chloromethyl)phenyl]-1,2-dimethyl-5-nitro-1*H*-imidazole (**1**).

4-[4-(Chloromethyl)phenyl]-1,2-dimethyl-5-nitro-1*H*-imidazole (**1**) was prepared from the previously described [4-(1,2-dimethyl-5-nitro-1*H*-imidazol-4-yl)phenyl]methanol [29], according to a classical chlorination reaction with SOCl_2_ [20] (Scheme 1).

**Scheme 2:**
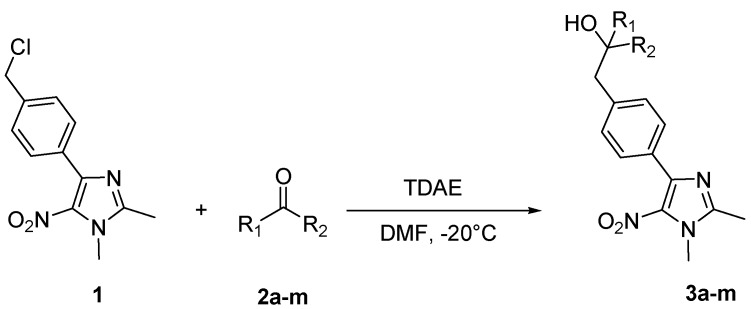
Reaction of **1** with various carbonyl compounds using TDAE strategy.

The reaction of **1** with 3 equivalents of various aromatic aldehydes **2a-j** in the presence of TDAE at –20 °C for 1 h, followed by 24 h at rt (Scheme 2), led to the corresponding alcohol derivatives **3a-j** in moderate to good yields (24–78%) as shown in Table 1. 

**Table 1:** Products’ yields from the reaction of **1** with various carbonyl compounds using TDAE strategy.

Carbonyl compound		Product ^a^	Product number	Yield (%) ^b^
**2a**	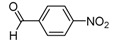	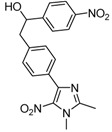	**3a**	69
**2b**	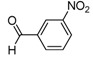	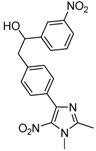	**3b**	46
**2c**	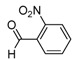	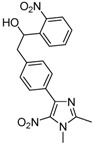	**3c**	37
**2d**	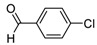	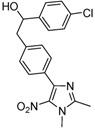	**3d**	24
**2e**	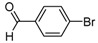	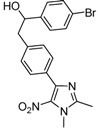	**3e**	60
**2f**	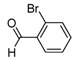	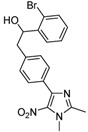	**3f**	68
**2g**	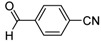	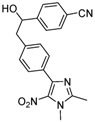	**3g**	78
**2h**	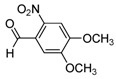	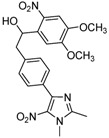	**3h**	30
**2i**	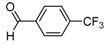	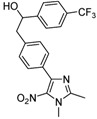	**3i**	45
**2j**	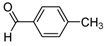	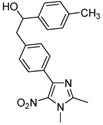	**3j**	25
**2k**	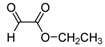	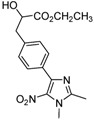	**3k**	42
**2l**	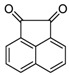	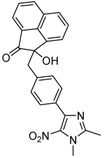	**3l**	45
**2m^c^**	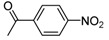	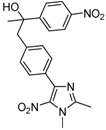	**3m**	64

^a^ All the reactions are performed using 3 equivalents of carbonyl compounds **2a-l**, 1 equivalent of chloride **1** and 1 equivalent of TDAE in anhydrous DMF stirred at –20 °C for 1 h and then warmed up to rt for 24 h. ^b^ % Yield relative to chloride **1**. ^c^ The reaction is performed using 3 equivalents of carbonyl compounds **2m**, 1 equivalent of chloride **1** and 1 equivalent of TDAE in anhydrous DMF stirred at –20 °C for 1 h and then warmed up to 80 °C for 24 h.

High yields were obtained with *p*-nitrobenzaldehyde (**2a**), *o*-bromobenzaldehyde (**2f**) and *p*-cyanobenzaldehyde (**2g**), whereas *p*-chlorobenzaldehyde (**2d**), *p*-methylbenzaldehyde (**2j**) and 6-nitroveratraldehyde (**2h**) produced low yields. In summary, the difference of yields seems explained by the electronic effects, whereby the electron-withdrawing groups furnished the best yields, the electron-donating groups furnished the lowest yields and the halogen groups led to varied yields. The different yields with halobenzaldehydes could be explained by some purification problems encountered with the compound **3d**. With nitrobenzaldehydes, steric hindrance could explain the difference between the *o*- and *m*-nitrobenzaldehyde (37% *versus* 46%). Moreover, after the reaction with aromatic aldehydes, we have investigated the reaction of **1** with α-keto-ester derivatives such as ethyl glyoxylate (**2k**), with an α-diketone such as acenaphthenedione (**2l**) and with a ketone like *p*-nitroacetophenone (**2m**). The reaction of **1** in the presence of TDAE with these electrophiles furnished the corresponding hydroxyl derivatives **3k-m** in moderate to good yields (42–64%), as shown in Table 1. As observed in the *p*-nitrobenzyl series [19], due to its poor reactivity, *p*-nitroacetophenone (**2l**) needed heating for 24 h at 80 °C to drive the reaction to completion.

## 3. Experimental

Melting points were determined on Büchi B-540 and are uncorrected. Elemental analyses were carried out at the Spectropole, Faculté des Sciences et Techniques de Saint-Jérome. Both ^1^H- and ^13^C-NMR spectra were determined on Bruker ARX 200 spectrometer. The ^1^H chemical shifts were reported as parts per million downfield from tetramethylsilane (Me_4_Si), and the ^13^C chemical shifts were referenced to the solvent peak of CDCl_3_ (76.9 ppm). The following adsorbent was used for column chromatography: silica gel 60 (Merck, 230–400 mesh). Thin-layer chromatography was performed with silica gel Merck 60F-254 (0.25 mm layer thickness). 

*4-[4-(Chloromethyl)phenyl]-1,2-dimethyl-5-nitro-1H-imidazole* (**1**): This compound was prepared from [4-(1,2-dimethyl-5-nitro-1*H*-imidazol-4-yl)phenyl]methanol [29], according to a classical chlorination reaction with SOCl_2_ [20]. Yellow solid; mp 120 °C (isopropyl alcohol); ^1^H-NMR (CDCl_3_) δ: 2.51 (s, 3H, CH_3_), 3.90 (s, 3H, CH_3_), 4.62 (s, 2H, CH_2_), 7.45 (d, *J* = 8.1 Hz, 2H, 2xCH), 7.76 (d, *J* = 8.1 Hz, 2H, 2xCH); ^13^C-NMR (CDCl_3_) δ: 14.0 (CH_3_), 34.2 (CH_3_), 45.8 (CH_2_), 128.2 (2xCH), 129.9 (2xCH), 131.6 (C), 138.6 (C), 142.5 (C), 148.3 (C). The C-nitro was not observed in this experiment; Anal. Calcd for C_12_H_12_N_3_O_2_Cl: C, 54.25; H, 4.55; N, 15.82. Found: C, 54.31; H, 4.61; N, 15.97.

*General Procedure for the Reaction of Chloride* **1*** and Aromatic Carbonyl Derivatives* **2a-m** *Using TDAE*

A solution of 4-[4-(chloromethyl)phenyl]-1,2-dimethyl-5-nitro-1*H*-imidazole (**1**, 0.15 g, 0.56 mmol, 1 equiv.) in anhydrous DMF (6 mL) and the corresponding carbonyl derivative **2a-m** (1.68 mmol, 3 equiv.) were placed under nitrogen at −20 °C in a two-necked flask equipped with a silica-gel drying tube and a nitrogen inlet. The solution was stirred and kept at this temperature for 30 min and then the TDAE (0.11 g, 0.56 mmol, 1 equiv.) was added dropwise via a syringe. The solution was vigorously stirred at −20 °C for 1 h and then warmed up to room temperature for 24 h [heating for 24 h at 80 °C in the case of *p*-nitroacetophenone (**2m**)]. After this time TLC analysis (CH_2_Cl_2_/EtOAc (7/3) as eluent) clearly showed that **1** was totally consumed. The orange-red turbid solution was filtered and rinsed with toluene. The organic layer was washed with H_2_O (3 × 40 mL) and dried over MgSO_4_. Evaporation of the solvent left an orange viscous liquid as crude product. Purification by silica gel chromatography [CH_2_Cl_2_/EtOAc (7:3)] gave the corresponding arylethanol or α-hydroxyester derivatives.

*2-[4-(1,2-Dimethyl-5-nitro-1H-imidazol-4-yl)phenyl]-1-(4-nitrophenyl)ethanol* (**3a**): Beige solid; mp 200 °C (isopropyl alcohol); ^1^H-NMR (CDCl_3_) δ: 2.60 (s, 3H, CH_3_), 3.02–3.10 (m, 2H, CH_2_), 3.95 (s, 3H, CH_3_), 5.04 (dd, *J* = 5.3 Hz et *J* = 7.9 Hz, 1H, CH), 7.26 (d, *J* = 8.0 Hz, 2H, 2xCH), 7.52 (d, *J* = 8.7 Hz, 2H, 2xCH), 7.73 (d, *J* = 8.0 Hz, 2H, 2xCH), 8.20 (d, *J* = 8.7 Hz, 2H, 2xCH); ^13^C-NMR (CDCl_3_) δ: 13.9 (CH_3_), 34.1 (CH_3_), 45.1 (CH_2_), 72.7 (CH), 123.3 (2xCH), 127.3 (2xCH), 128.9 (2xCH), 129.3 (2xCH), 129.9 (C), 139.7 (C), 142.3 (C), 146.6 (C), 149.2 (C), 153.7 (C). The C-nitro was not observed in this experiment; Anal. Calcd for C_19_H_18_N_4_O_5_: C, 59.68; H, 4.74; N, 14.65. Found: C, 59.68; H, 4.92; N, 14.41.

*2-[4-(1,2-Dimethyl-5-nitro-1H-imidazol-4-yl)phenyl]-1-(3-nitrophenyl)ethanol* (**3b**): Yellow solid; mp 225 °C (ethyl alcohol); ^1^H-NMR (DMSO-d_6_) δ: 2.45 (s, 3H, CH_3_), 2.94–2.99 (m, 2H, CH_2_), 3.81 (s, 3H, CH_3_), 4.96–5.04 (m, 1H, CH), 5.67–5.69 (m, 1H, OH), 7.27 (d, *J* = 8.2 Hz, 2H, 2xCH), 7.58 (d, *J* = 8.2 Hz, 2H, 2xCH), 7.64 (s, 1H, CH), 7.79 (d, *J* = 7.7 Hz, 1H, CH), 8.09 (d, *J* = 8.2 Hz, 1H, CH), 8.22 (s, 1H, CH); ^13^C-NMR (DMSO-d_6_) δ: 13.9 (CH_3_), 34.1 (CH_3_), 45.2 (CH_2_), 72.5 (CH), 120.7 (CH), 121.9 (CH), 128.9 (2xCH), 129.3 (2xCH), 129.6 (CH), 130.1 (C), 132.9 (CH), 134.6 (C), 139.8 (C), 142.4 (C), 147.8 (C), 148.2 (C), 149.2 (C); Anal. Calcd for C_19_H_18_N_4_O_5_: C, 59.68; H, 4.74; N, 14.65. Found: C, 59.25; H, 4.78; N, 14.36.

*2-[4-(1,2-Dimethyl-5-nitro-1H-imidazol-4-yl)phenyl]-1-(2-nitrophenyl)ethanol* (**3c**): Orange solid; mp 216 °C (ethyl alcohol); ^1^H-NMR (DMSO-d_6_) δ: 2.45 (s, 3H, CH_3_), 2.80 (dd, *J* = 8.1 Hz et *J* = 13.0 Hz, 1H, CH_2_), 3.04 (dd, *J* = 8.1 Hz and *J* = 13.0 Hz, 1H, CH_2_), 3.82 (s, 3H, CH_3_), 5.25 (m, 1H, CH), 5.66 (d, *J* = 4.9 Hz, 1H, OH), 7.31 (d, *J* = 8.1 Hz, 2H, 2xCH), 7.53 (t, *J* = 8.1 Hz, 1H, CH), 7.63 (d, *J* = 8.1 Hz, 2H, 2xCH), 7.75 (t, *J* = 8.1 Hz, 1H, CH), 7.86 (d, *J* = 8.1 Hz, 1H, CH), 7.94 (d, *J* = 8.1 Hz, 1H, CH); ^13^C-NMR (DMSO-d_6_) δ: 14.0 (CH_3_), 34.1 (CH_3_), 44.7 (CH_2_), 69.2 (CH), 124.0 (CH), 128.4 (CH), 128.5 (CH), 129.1 (4xCH), 130.2 (C), 133.6 (CH), 134.7 (C), 140.2 (C), 140.8 (C), 142.5 (C), 147.5 (C), 149.3 (C); Anal. Calcd for C_19_H_18_N_4_O_5_: C, 59.68; H, 4.74; N, 14.65. Found: C, 58.83; H, 4.82; N, 14.01.

*1-(4-Chlorophenyl)-2-*[4-(1,2-dimethyl-5-nitro-1H-imidazol-4-yl)phenyl]*ethanol* (**3d**): Pink solid; mp 199 °C (isopropyl alcohol); ^1^H-NMR (DMSO-d_6_): 2.45 (s, 3H, CH_3_), 2.90 (d, *J* = 6.6 Hz, 2H, CH_2_), 3.81 (s, 3H, CH_3_), 4.77–4.85 (m, 1H, CH), 5.44 (d, *J* = 4.8 Hz, 1H, OH), 7.23 (d, *J* = 8.1 Hz, 2H, 2xCH), 7.35 (bs, 4H, 4xCH), 7.57 (d, *J* = 8.1 Hz, 2H, 2xCH); ^13^C-NMR (DMSO-d_6_) δ: 13.9 (CH_3_), 34.1 (CH_3_), 45.4 (CH_2_), 72.9 (CH), 128.0 (4xCH), 128.9 (2xCH), 129.2 (2xCH), 130.0 (C), 131.3 (C), 134.6 (C), 140.2 (C), 142.5 (C), 144.8 (C), 149.3 (C); Anal. Calcd for C_19_H_18_ClN_3_O_3_: C, 61.38; H, 4.88; N, 11.30. Found: C, 60.85; H, 4.94; N, 11.03.

*1-(4-Bromophenyl)-2-[4-(1,2-dimethyl-5-nitro-1H-imidazol-4-yl)phenyl]ethanol* (**3e**): White solid; mp 211 °C (isopropyl alcohol); ^1^H-NMR (DMSO-d_6_) δ: 2.45 (s, 3H, CH_3_), 2.90 (d, *J* = 6.5 Hz, 2H, CH_2_), 3.81 (s, 3H, CH_3_), 4.80 (q, *J* = 5.3 Hz, 1H, CH), 5.42 (d, *J* = 4.8 Hz, 1H, OH), 7.23 (d, *J* = 8.2 Hz, 2H, 2xCH), 7.29 (d, *J* = 8.4 Hz, 2H, 2xCH), 7.49 (d, *J* = 8.4 Hz, 2H, 2xCH), 7.57 (d, *J* = 8.2 Hz, 2H, 2xCH); ^13^C-NMR (DMSO-d_6_) δ: 13.9 (CH_3_), 34.1 (CH_3_), 45.3 (CH_2_), 72.9 (CH), 119.8 (C), 128.4 (2xCH), 128.9 (2xCH), 129.2 (2xCH), 130.0 (C), 130.9 (2xCH), 134.6 (C), 140.2 (C), 142.5 (C), 145.2 (C), 149.3 (C); Anal. Calcd for C_19_H_18_BrN_3_O_3_: C, 54.82; H, 4.36; N, 10.09. Found: C, 54.78; H, 4.42; N, 9.97.

*1-(2-Bromophenyl)-2-[4-(1,2-dimethyl-5-nitro-1H-imidazol-4-yl)phenyl]ethanol* (**3f**): Yellow solid; mp 228 °C (isopropyl alcohol); ^1^H-NMR (DMSO-d_6_) δ: 2.45 (s, 3H, CH_3_), 2.59 (dd, *J* = 9.0 Hz et *J* = 13.7 Hz, 1H, CH_2_), 2.98 (dd, *J* = 9.0 Hz and *J* = 13.7 Hz, 1H, CH_2_), 3.82 (s, 3H, CH_3_), 5.00–5.09 (m, 1H, CH), 5.56 (d, *J* = 4.9 Hz, 1H, OH), 7.20 (t, *J* = 7.7 Hz, 1H, CH), 7.32 (d, *J* = 8.0 Hz, 2H, 2xCH), 7.41 (t, *J* = 7.7 Hz, 1H, CH), 7.56 (s, 1H, CH), 7.62 (d, *J* = 8.0 Hz, 3H, 3xCH); ^13^C-NMR (DMSO-d_6_) δ: 13.9 (CH_3_), 34.1 (CH_3_), 43.9 (CH_2_), 72.6 (CH), 121.3 (C), 127.9 (CH), 128.0 (CH), 129.0 (5xCH), 130.1 (C), 132.3 (CH), 134.7 (C), 140.4 (C), 142.5 (C), 144.7 (C), 149.3 (C); Anal. Calcd for C_19_H_18_BrN_3_O_3_: C, 54.82; H, 4.36; N, 10.09. Found: C, 54.70; H, 4.44; N, 9.95.

*4-{2-[4-(1,2-Dimethyl-5-nitro-1H-imidazol-4-yl)phenyl]-1-hydroxyethyl}benzonitrile* (**3g**): Yellow solid; mp 208 °C (isopropyl alcohol); ^1^H-NMR (DMSO-d_6_) δ: 2.45 (s, 3H, CH_3_), 2.91-2.92 (m, 2H, CH_2_), 3.81 (s, 3H, CH_3_), 4.87–4.96 (m, 1H, CH), 5.60 (d, *J* = 4.9 Hz, 1H, OH), 7.24 (d, *J* = 8.1 Hz, 2H, 2xCH), 7.56 (t, *J* = 8.1 Hz, 4H, 4xCH), 7.77 (d, *J* = 8.1 Hz, 2H, 2xCH); ^13^C-NMR (DMSO-d_6_) δ: 13.9 (CH_3_), 34.1 (CH_3_), 45.1 (CH_2_), 73.0 (CH), 109.6 (C), 119.2 (C), 127.1 (2xCH), 128.9 (2xCH), 129.2 (2xCH), 130.1 (C), 132.1 (2xCH), 134.6 (C), 139.8 (C), 142.5 (C), 149.3 (C), 151.6 (C); Anal. Calcd for C_20_H_18_N_4_O_3_: C, 66.29; H, 5.01; N, 15.46. Found: C, 66.17; H, 5.07; N, 15.22.

*1-(4,5-Dimethoxy-2-nitrophenyl)-2-[4-(1,2-dimethyl-5-nitro-1H-imidazol-4-yl)phenyl]ethanol* (**3h**): Black solid; mp 234 °C (acetonitrile); ^1^H-NMR (DMSO-d_6_) δ: 2.46 (s, 3H, CH_3_), 2.81 (dd, *J* = 7.9 Hz et *J* = 13.4 Hz, 1H, CH_2_), 3.05 (dd, *J* = 2.1 Hz and *J* = 13.4 Hz, 1H, CH_2_), 3.83 (s, 3H, CH_3_), 3.84 (s, 3H, CH_3_), 3.86 (s, 3H, CH_3_), 5.37–5.45 (m, 1H, CH), 5.61 (d, *J* = 4.9 Hz, 1H, OH), 7.29–7.34 (m, 3H, 3xCH), 7.59–7.65 (m, 3H, 3xCH); ^13^C-NMR (DMSO-d_6_) δ: 14.0 (CH_3_), 34.1 (CH_3_), 44.5 (CH_2_), 56.2 (2xCH_3_), 69.1 (CH), 107.5 (CH), 109.9 (CH), 129.0 (2xCH), 129.2 (2xCH), 130.2 (C), 134.7 (C), 136.8 (C), 139.1 (C), 140.3 (C), 142.6 (C), 147.4 (C), 149.3 (C), 153.3 (C); Anal. Calcd for C_21_H_22_N_4_O_7_: C, 57.01; H, 5.01; N, 12.66. Found: C, 56.81; H, 4.95; N, 12.50.

*2-[4-(1,2-Dimethyl-5-nitro-1H-imidazol-4-yl)phenyl]-1-[4-(trifluoromethyl)phenyl]ethanol* (**3i**): Yellow solid; mp 195 °C (isopropyl alcohol); ^1^H-NMR (CDCl_3_) δ: 2.51 (s, 3H, CH_3_), 3.01–3.06 (m, 2H, CH_2_), 3.91 (s, 3H, CH_3_), 4.96 (dd, *J* = 5.5 Hz and *J* = 7.8 Hz, 1H, CH), 7.24 (d, *J* = 7.8 Hz, 2H, 2xCH), 7.46 (d, *J* = 8.2 Hz, 2H, 2xCH), 7.60 (d, *J* = 8.2 Hz, 2H, 2xCH), 7.70 (d, *J* = 8.2 Hz, 2H, 2xCH); ^13^C-NMR (CDCl_3_) δ: 13.9 (CH_3_), 34.1 (CH_3_), 45.3 (CH_2_), 73.0 (CH), 124.6 (q, *J* = 272 Hz, C), 124.9 (q, *J* = 3.5 Hz, CH), 125.0 (CH), 126.9 (2xCH), 127.6 (q, *J* = 31.5 Hz, C), 129.0 (2xCH), 129.2 (2xCH), 130.0 (C), 134.7 (C), 140.1 (C), 142.5 (C), 149.3 (C), 150.6 (C); Anal. Calcd for C_20_H_18_F_3_N_3_O_3_: C, 59.26; H, 4.48; N, 10.37. Found: C, 59.26; H, 4.81; N, 10.30.

*2-[4-(1,2-Dimethyl-5-nitro-1H-imidazol-4-yl)phenyl]-1-p-tolylethanol* (**3j**): Yellow solid; mp 155 °C (isopropyl alcohol); ^1^H-NMR (CDCl_3_) δ: 2.16 (bs, 1H, OH), 2.34 (s, 3H, CH_3_), 2.50 (s, 3H, CH_3_), 3.05 (d, *J* = 6.7 Hz, 2H, CH_2_), 3.89 (s, 3H, CH_3_), 4.88 (t, *J* = 6.7 Hz, 1H, CH), 7.14 (d, *J* = 8.0 Hz, 2H, 2xCH), 7.25 (d, *J* = 8.0 Hz, 4H, 4xCH), 7.69 (d, *J* = 8.0 Hz, 2H, 2xCH); ^13^C-NMR (CDCl_3_) δ: 14.1 (CH_3_), 21.1 (CH_3_), 34.1 (CH_3_), 45.9 (CH_2_), 75.0 (CH), 125.8 (2xCH), 129.1 (2xCH), 129.2 (2xCH), 129.6 (2xCH), 130.0 (C), 134.7 (C), 137.2 (C), 139.7 (C), 140.8 (C), 143.5 (C), 148.3 (C); Anal. Calcd for C_20_H_21_N_3_O_3_: C, 68.36; H, 6.02; N, 11.96. Found: C, 68.36; H, 6.16; N, 11.90.

*Ethyl 3-[4-(1,2-dimethyl-5-nitro-1H-imidazol-4-yl)phenyl]-2-hydroxypropanoate* (**3k**): Yellow solid; mp 99 °C (isopropyl alcohol); ^1^H-NMR (CDCl_3_) δ: 1.28 (t, *J* = 7.1 Hz, 3H, CH_3_), 2.55 (s, 3H, CH_3_), 3.02 (dd, *J* = 6.7 Hz and *J* = 13.9 Hz, 1H, CH_2_), 3.17 (dd, *J* = 4.6 Hz and *J* = 13.9 Hz, 1H, CH_2_), 3.91 (s, 3H, CH_3_), 4.22 (q, *J* = 7.1 Hz, 2H, CH_2_), 4.46 (dd, *J* = 4.6 Hz and *J* = 6.7 Hz, 1H, CH), 7.31 (d, *J* = 7.9 Hz, 2H, 2xCH), 7.72 (d, *J* = 8.2 Hz, 2H, 2xCH); ^13^C-NMR (CDCl_3_) δ: 14.2 (CH_3_), 34.5 (CH_3_), 40.4 (CH_2_), 61.9 (CH_2_), 70.9 (CH_3_), 77.2 (CH), 126.8 (C), 129.6 (2xCH), 129.8 (2xCH), 134.1 (C), 139.4 (C), 139.8 (C), 147.7 (C), 173.9 (C); Anal. Calcd for C_16_H_19_N_3_O_5_: C, 57.65; H, 5.75; N, 12.61. Found: C, 57.74; H, 5.87; N, 12.65.

*2-[4-(1,2-Dimethyl-5-nitro-1H-imidazol-4-yl)benzyl]-2-hydroxyacenaphthylen-1(2H)-one* (**3l**): Yellow solid; mp 211 °C (isopropyl alcohol); ^1^H-NMR (DMSO-d_6_) δ: 2.41 (s, 3H, CH_3_), 3.17 (d, *J* = 13.2 Hz, 1H, CH_2_), 3.41 (d, *J* = 13.2 Hz, 1H, CH_2_), 3.77 (s, 3H, CH_3_), 6.99 (d, *J* = 7.5 Hz, 2H, 2xCH), 7.12 (bs, 1H, OH), 7.33–7.40 (m, 3H, 3xCH), 7.63–7.77 (m, 2H, 2xCH), 7.84 (d, *J* = 8.4 Hz, 1H, CH), 7.94 (d, *J* = 7.0 Hz, 1H, CH), 8.19 (d, *J* = 8.1 Hz, 1H, CH); ^13^C-NMR (DMSO-d_6_) δ: 13.7 (CH_3_), 34.2 (CH_3_), 42.9 (CH_2_), 79.8 (C), 121.3 (2xCH), 125.1 (CH), 128.5 (2xCH), 128.7 (2xCH), 129.9 (CH), 130.0 (2xCH), 130.2 (C), 131.0 (C), 132.0 (C), 134.6 (C), 137.0 (C), 140.4 (C), 140.8 (C), 141.7 (C), 149.1 (C), 205.3 (C); Anal. Calcd for C_24_H_19_N_3_O_4_: C, 69.72; H, 4.63; N, 10.16. Found: C, 69.41; H, 4.73; N, 10.13.

*1-[4-(1,2-Dimethyl-5-nitro-1H-imidazol-4-yl)phenyl]-2-(4-nitrophenyl)propan-2-ol* (**3m**): Orange solid; mp 179 °C (acetonitrile); ^1^H-NMR (CDCl_3_) δ: 1.60 (s, 3H, CH_3_), 2.51 (s, 3H, CH_3_), 3.12 (dd, *J* = 13.2 Hz and *J* = 18.3 Hz, 2H, CH_2_), 3.90 (s, 3H, CH_3_), 7.06 (d, *J* = 8.3 Hz, 2H, 2xCH), 7.57 (d, *J* = 9.0 Hz, 2H, 2xCH), 7,63 (d, *J* = 8.3 Hz, 2H, 2xCH), 8.16 (d, *J* = 9.0 Hz, 2H, 2xCH); ^13^C-NMR (CDCl_3_) δ: 14.0 (CH_3_), 29.4 (CH_3_), 34.1 (CH_3_), 50.0 (CH_2_), 74.4 (C), 123.3 (2xCH), 126.1 (2xCH), 129.4 (2xCH), 130.2 (2xCH), 130.4 (C), 134.7 (C), 137.2 (C), 142.9 (C), 146.7 (C), 148.3 (C), 154.8 (C); Anal. Calcd for C_20_H_20_N_4_O_5_: C, 60.60; H, 5.09; N, 14.13. Found: C, 60.60; H, 5.15; N, 13.97.

## 4. Conclusions

We have reported the synthesis of substituted 2-[4-(1,2-dimethyl-5-nitro-1*H*-imidazol-4-yl)phenyl]-1-arylethanols, ethyl 3-[4-(1,2-dimethyl-5-nitro-1*H*-imidazol-4-yl)phenyl]-2-hydroxypropanoate and 2-[4-(1,2-dimethyl-5-nitro-1*H*-imidazol-4-yl)benzyl]-2-hydroxy-acenaphthylen-1(2*H*)-one from the reaction of 4-[4-(chloromethyl)phenyl]-1,2-dimethyl-5-nitro-1*H*-imidazole with various aromatic carbonyl and α-carbonyl ester derivatives using TDAE methodology. We have shown a new application of TDAE methodology in a heterocyclic series. The pharmacological evaluation, against metronidazole-resistant lines of *Blastocystis sp.*, of all synthesized compounds is under active investigation. 
